# Intraocular lens power calculation in pediatric cataract surgery: A narrative review

**DOI:** 10.1097/MD.0000000000042072

**Published:** 2025-04-04

**Authors:** Bogumiła Wójcik-Niklewska, Martyna Nocoń-Bratek, Klaudia Szala

**Affiliations:** a Department of Pediatric Ophthalmology, Faculty of Medical Sciences in Katowice, Medical University of Silesia, Poland; b Professor Kornel Gibiński University Hospital Center, Medical University of Silesia, Katowice, Poland; c Department of Ophtalmology, Faculty of Medical Sciences in Katowice, Medical University of Silesia, Poland; d Department of Ophthalmology, Students’ Scientific Society, Faculty of Medical Sciences in Katowice, Medical University of Silesia, Katowice, Poland.

**Keywords:** aphakia, children, congenital cataract, intraocular lens, pseudophakia

## Abstract

Congenital cataract is a lens opacification that disrupts normal visual development, requiring early surgical intervention to prevent amblyopia. In children, timely surgery, followed by optical correction and vision rehabilitation, is crucial for achieving binocular vision with foveal fixation. The recommended surgical timing is within 8 weeks for unilateral cases and by 4 months for bilateral cases to minimize long-term visual impairment. Despite advancements in intraocular lens (IOL) technology and ophthalmic microsurgery, accurate IOL power selection in pediatric patients remains a challenge due to axial length growth, biometric variability, and the reliance on formulas derived from adult models. These factors contribute to postoperative refractive errors, making proper formula selection essential in minimizing additional corrective interventions. Traditional third-generation formulas, such as the Sanders–Retzlaff–Kraff-T and Holladay 1, are commonly used in pediatric cases. However, recent studies suggest that Barrett Universal II offers greater accuracy in older children, owing to its advanced vergence-based algorithm and improved axial length prediction. Emerging formulas, including Hill-RBF 3.0 and Kane, show promise but require further validation in pediatric cohorts. Additionally, ocular growth dynamics must be accounted for when determining postoperative refractive targets. Younger children often require undercorrection to compensate for axial elongation, and biometric formulas must be chosen accordingly to optimize long-term outcomes. The lack of pediatric-specific formulas further complicates IOL selection, emphasizing the need for new models that integrate machine learning algorithms and growth prediction data.

## 1. Introduction

Cataract is a pathological disorder of the lens involving partial or complete opacity, resulting in loss of transparency and optical properties. It requires surgical extraction, after which an intraocular lens (IOL) may be implanted, or the eye remains aphakic, necessitating optical correction.^[1]^ Paediatric cataracts are one of the leading causes of reduced visual acuity in children, accounting for up to 10% of blindness in global pediatric population. Its prevalence is 4.24 per 10,000 live births.^[2]^ Paediatric cataracts may be congenital or acquired during postnatal period.^[3]^ Congenital cataract (CC) occurs already at birth or is diagnosed in first years of a child’s life; its location and morphological appearance can differ significantly. Aetiology of pediatric cataracts varies considerably, examples being chromosomal abnormalities (Down syndrome), genetic conditions, systemic diseases and syndromes (renal syndromes), metabolic disorders (diabetes), intrauterine infections (TORCH syndrome), and ocular anomalies (aniridia, microphthalmia, persistent fetal vasculature).^[4,5]^ Cataracts may also result from exposure to radiation or corticosteroid therapy, as well as from trauma or in association with uveitis.^[6]^ Heredity remains the most common etiology of CCs.^[4]^ In case of opacities that prevent normal development of visual processes, surgery is only treatment option. Unilateral cataracts should be removed before child is 8 weeks old; optimum age is 4 to 6 weeks.^[7]^ In case of bilateral cataracts, surgery should be performed before appearance of strabismus or nystagmus,^[8]^ that is, by end of 10th week of age with an interval of 7 to 14 days between eyes^[4]^ or in case of bilateral visually significant cataracts surgery should be performed between 6 to 8 weeks of age.^[7]^ However, performing surgery at an earlier stage increases risk of developing another serious vision-threatening condition, glaucoma, which involves progressive optic nerve damage due to elevated intraocular pressure.^[9]^ Reports indicate that incidence of postoperative glaucoma ranges up to 32%.^[10–13]^

Post-traumatic cataracts, especially in children under 6 years of age, should be extracted within a few weeks after injury.^[2]^ Developmental cataracts, which emerge in early childhood rather than at birth, can also occur. In some cases, surgery may be postponed if visual development remains unaffected; however, in all instances of visually significant cataracts, substantial visual impairment is unavoidable without surgical intervention. Certain types, such as lamellar or posterior cataracts, may advance rapidly and necessitate early surgical intervention.^[6]^ As every surgery procedure, cataract removal carries complications. Short-term complications may include intraoperative and early postoperative risks such as bleeding, inflammation or infection.^[6,14,15]^ Additionally, children must undergo general anesthesia, which carries its own risks. Long-term complications can develop over time following surgery and may include posterior capsular opacification, secondary membrane formation, lens material reproliferation, glaucoma, IOL dislocation (if implanted), retinal detachment, and potential vision loss.^[6,16]^ In some cases, additional surgical procedures may be required to manage these complications, with posterior capsule opacification (PCO) being most common reason for reoperation. This condition can also contribute to development of amblyopia.^[17]^

Beyond surgical risks, visual rehabilitation plays a critical role in achieving successful outcomes. Even with a well-performed surgery, amblyopia can develop if appropriate postoperative care, such as optical correction and patching therapy, is not maintained. In infants, CCs that significantly impair vision must be addressed promptly to prevent onset of irreversible deprivation amblyopia.^[6]^ Adherence to follow-up care and rehabilitation programs is crucial for optimizing long-term visual function. Additionally, traditional IOL power calculation formulas, originally developed for adult eyes, often yield suboptimal predictions in pediatric patients, necessitating alternative approaches or modifications.^[7]^ Despite advancements in surgical techniques and postoperative management, literature remains inconclusive regarding optimal IOL power calculation strategy for infants and young children. This review aims to explore the existing literature on IOL power calculation in pediatric cataract surgery, highlighting challenges, limitations and potential solutions to improve refractive outcomes and long-term visual prognosis.

## 2. Methods

A comprehensive literature search identified relevant studies on IOL power calculation in pediatric cataract surgery. The search included original research articles, systematic reviews, clinical trials, and case reports related to IOL formulas, biometric techniques, and postoperative outcomes in children. Databases searched included PubMed/MEDLINE, Scopus, Web of Science, Cochrane Library, StatPearls, ResearchGate, and Google Scholar for gray literature. A combination of keywords and Medical Subject Headings was used, including “pediatric cataract,” “intraocular lens calculation,” “biometry in children,” “IOL power formulas,” “myopic shift,” “aphakia correction,” and “visual outcomes.” Boolean operators such as AND and OR refined the search, for example: (“pediatric cataract” AND “IOL power calculation”) OR (“biometry in children” AND “myopic shift”).

Inclusion criteria covered studies published between 2000 and 2024, articles in English, research focused on pediatric populations aged 0 to 18 years, and studies addressing IOL power calculation formulas, biometric measurements, or postoperative refractive outcomes. Exclusion criteria involved non-English publications, animal studies, research focused solely on adults, and letters, editorials, or opinion pieces without primary data. Extracted data were synthesized qualitatively to provide an overview of current practices and evidence in pediatric IOL power calculation.

## 3. Correction of aphakia

Following cataract surgery, it is essential to correct the visual defect in the child’s aphakic eye and implement regular vision rehabilitation. Aphakia refers to the absence of the natural crystalline lens, disrupting the eye’s refractive properties and necessitating optical correction.^[18]^ The goal is to improve visual acuity and support the proper development of binocular single vision with foveal fixation. Binocular single vision enables both eyes to work together, allowing the perception of a single image. This process relies on proper bifoveal fixation and normal retinal correspondence. Disruptions can lead to binocular vision abnormalities.^[19]^ Foveal fixation, involving brief focus on targets using the fovea, the central part of the retina responsible for the sharpest vision, is crucial for this mechanism. The fovea also serves as the reference point for the oculomotor system, ensuring precise eye movement control, essential for maintaining optimal binocular vision.^[20]^

Significant visual impairment, including strabismus and nystagmus, can arise from improper binocular vision and inadequate foveal fixation. This is especially critical in early infancy, during the third stage of foveal development, which involves the packing and differentiation of foveal cone photoreceptors.^[21]^ This process, triggered and maintained by light stimulation, facilitates visual image formation, albeit initially blurred. Insufficient light exposure and visual input can interfere with cone packing, potentially preventing high-resolution vision development even after lens opacity removal.^[21]^ The impact of early developmental processes underscores the importance of effective visual rehabilitation following pediatric cataract surgery. Both the degree of preexisting visual impairment and the rehabilitation strategy influence the final visual outcome.^[22]^

If a primary IOL is not implanted, aphakia is corrected with spectacles or contact lenses.^[2]^ The loss of the eye’s primary focusing mechanism results in severe hyperopia, reduced visual clarity, and impaired depth perception, leading to developmental delays if uncorrected.^[23]^ Optical aid power is adjusted to the eyeball’s growth rate. Mutti et al observed an average axial length (AL) growth of 1.2 ± 0.51 mm between 3 and 9 months of age.^[24]^ Bauch et al demonstrated that both eyes exhibit comparable growth patterns, with the most rapid AL expansion occurring within the first 10 months of life, followed by a slower growth rate.^[25]^ Axial length undergoes significant elongation in early childhood, gradually decelerating over time.^[26,27]^

These continuous ocular changes pose challenges in determining intraocular lens power, as future refractive shifts depend on AL growth, anterior segment development, and corneal power. Predicting these changes is crucial for optimal long-term visual outcomes, as miscalculations can lead to refractive errors requiring further correction.^[28–30]^ Contact lenses for aphakia require proper wear to prevent corneal complications.^[31]^ Eyeglasses are unsuitable when anisometropia exceeds 3.00 diopters (D) of refractive error or 1.50 D of astigmatism due to the high risk of aniseikonia.^[32]^ Normal eye development depends on coordinated changes in corneal and crystalline lens power as well as AL. The absence of the crystalline lens disrupts the developmental trajectory of the pediatric aphakic eye.^[24]^ Unilateral aphakia almost always results in anisometropia exceeding 3.00 D.^[33,34]^

One major challenge of using spectacles for unilateral aphakia is relative spectacle magnification, which hinders binocular vision and increases amblyopia risk. Additionally, high plus-power lenses introduce significant weight and bulk, making them impractical for newborns and infants. Beyond physical drawbacks, aphakic spectacles are often cosmetically, visually, and psychologically undesirable. Their high magnification can cause ring scotoma, distorted spatial perception, swim effects, and various optical aberrations such as chromatic, spherical, and coma aberrations, further complicating their use.^[35]^

Treatment outcomes depend on parental compliance and the child’s acceptance of the correction method. Spectacles may be considered in exceptional cases when parents are unable or unwilling to manage contact lens use.^[36]^ In unilateral aphakia, where contact lens intolerance is an issue, extensive vision rehabilitation is required, and secondary intraocular lens implantation is often the best solution.^[22]^

The Infant Aphakia Treatment Study (IATS) evaluated optical correction following cataract surgery in infants. Participants were randomly assigned to 1 of 2 groups: left aphakic with contact lenses or implanted with an IOL, with any remaining refractive error managed using spectacles.^[37,38]^ The findings showed that both treatment approaches allowed some patients to achieve good visual acuity and stereopsis.^[38]^

## 4. Intraocular lens implantation and calculating formulas

The development of modern ophthalmic surgery improves the quality of life for patients with cataracts. Selecting IOLs to compensate for aphakia depends on multiple factors, including the condition of the visual organ, the child’s age, and specific visual needs. In many pediatric aphakic eyes, the remaining lens capsule after cataract extraction is insufficient or exhibits adhesion and scarring between the anterior and PC, limiting the possibility of in-the-bag implantation. As a result, in such cases, the IOL can be positioned in the ciliary sulcus.^[39,40]^

A significant benefit of lens implantation is maintaining normal eyeball anatomy, restoring primary refraction, and preventing aniseikonia, which promotes binocular vision development.^[2]^ IOL implantation reduces dependency on external optical devices and provides constant refractive correction.^[41]^ In pediatric cataract surgery, selecting the appropriate IOL is crucial for addressing various visual needs. Monofocal IOLs typically provide clear distance vision; however, children may require additional spectacle correction for near tasks. Studies indicate that multifocal IOLs (mfIOLs) offer better near and intermediate vision than monofocal IOLs.^[42]^ However, several concerns must be addressed before their routine use. Proper centration and a round, stable pupil are essential for optimal function, yet studies indicate a significant risk of decentration and posterior synechiae, which may degrade multifocal optics and increase glare or contrast issues. Additionally, uncorrected corneal astigmatism as low as 1 D can impair image formation, further complicating their effectiveness in children.^[43]^ Given these considerations, monofocal IOLs remain the more extensively studied and safer option in pediatric populations.^[43]^

Implantation of the artificial lens can be primary, that is, simultaneous with cataract surgery, or secondary, performed at any time after initial cataract extraction to correct aphakia. Posterior chamber IOLs are recommended for secondary implantation.^[8]^ Intraocular lenses are most often placed in the lens capsule following the removal of opacified masses. Implant insertion in the lens capsule is associated with the least risk of peri- and postoperative complications.^[3]^ PCO, resulting from postoperative proliferation of cells within the capsular bag, remains the most prevalent complication following cataract surgery with IOL implantation.^[44]^ This issue is particularly significant in pediatric cataract surgery, where its occurrence nears 100%.^[45–47]^

Capsular bag distension syndrome (CBDS) is characterized by accumulating clear or cloudy fluid within the capsular bag, leading to axial displacement of the IOL, manifesting as unexpected myopia and decreased visual acuity after cataract removal.^[48]^ In adults, CBDS has been reported in 0.73% to 1% of cases following cataract surgery, whereas it is rarely documented in pediatric patients.^[49,50]^ To our knowledge, CBDS in children has been reported only by Medsinge and Nischal^[51]^ in a 7-year-old girl with juvenile idiopathic arthritis who underwent cataract surgery with posterior capsule IOL (PCIOL) implantation and by Matalia et al^[52]^ in a 14-year-old boy with bilateral congenital glaucoma who developed a posterior subcapsular cataract and subsequently underwent lens aspiration with PCIOL implantation. The possibility of CBDS in pediatric patients should be considered in cases of poor retinoscopic reflex due to turbid fluid between the PCIOL and the PC during the late postoperative period, particularly in children with a history of multiple surgeries.^[52]^

Other potential postoperative complications include a temporary increase in intraocular pressure, which may occur secondary to postoperative hyphema. This condition can be triggered by factors such as iris trauma, postoperative inflammation, retained viscoelastic material, or a steroid-induced response. Additionally, decentration of the IOL is a possible postoperative issue.^[40]^

Sulcus implantation is associated with an increased risk of complications, including iris chafing, IOL decentration, chronic inflammation, pupillary block, secondary glaucoma, secondary membrane formation, and haptic erosion into the sclera.^[53]^

In the absence of adequate capsular support, advances in surgical techniques and IOL designs have led to various alternative approaches. These include implanting an IOL within the capsular bag using a capsular tension ring for stabilization. Other alternatives involve a scleral-fixated PCIOL, either with or without a capsular tension ring or segment, an iris-sutured PCIOL, or an anterior chamber iris-fixated IOL.^[54,55]^ Due to the risk of spontaneous displacement of the IOL resulting from disruption of the fixation suture over time in scleral-fixated PCIOL, this option is not recommended for pediatric patients.^[56]^ Anterior chamber implants contribute to several complications, including pupillary ectopia, adhesions, hyphema, uveitis, and even retinal detachment.^[2]^

Technological advancements in IOLs influence biocompatibility and play a role in either reducing or contributing to posterior lens capsule opacities.^[57]^ An acrylic posterior chamber, monofocal, one-piece biconvex aspheric lens with a posterior square edge design and zero-degree angulation in the anteroposterior plane is recommended for pediatric cataract surgery. This lens provides excellent visual clarity by reducing spherical aberration and enhancing contrast sensitivity. Its posterior square edge minimizes the risk of PCO, while the biocompatible acrylic material ensures patient comfort and reduces inflammatory responses, making it a well-tolerated IOL option. The zero-degree angulation provides stable positioning, preventing decentration or rotation. Its one-piece construction facilitates implantation and ensures long-term stability, making it a reliable choice for monofocal intraocular implantation.^[58]^

Foldable lenses allow placement in the eyeball using an injector.^[2,59]^ Short-term observations by Pavlovic suggest that silicone implants are biocompatible with the child’s eye.^[59]^ MfIOLs and those with an extended depth of focus have been designed to compensate for the loss of accommodation after cataract surgery, resulting in spectacle independence.^[60,61]^ A greater depth of focus enables clear vision over a wider range of distances, reducing the need for additional corrective eyewear.^[61]^ Manufacturers recommend implanting these lenses in both eyes^[2]^ for optimal benefit. A small percentage of adult cataract surgery patients with a multifocal lens implant report adverse subjective visual phenomena (haloes, glare) or reduced contrast sensitivity, especially under mesopic conditions.^[62]^ Some case reports describe mfIOL use in older children (>4 years) with unilateral cataracts as an alternative to monofocal implantation.^[63]^

Despite the increasing number of IOL implantations in children, selecting the appropriate lens remains challenging due to AL growth and the lack of a universally accurate biometric formula for pediatric eyes.^[56]^ A significant difficulty lies in calculating implant power without cooperation from the child, often necessitating measurements under general anesthesia. Additionally, the calculation should consider the visual impairment history of the patient’s parents and siblings, as genetic factors may influence refractive development.

To address these challenges, several biometric formulas have been developed, though none are entirely ideal for pediatric eyes due to the dynamic nature of ocular growth. The power of the intraocular implant is calculated based on the child’s age, keratometry, and length of the ocular axis. Third-generation formulas such as The Sanders–Retzlaff–Kraff (SRK)-T, SRK II, Holladay I and II, Hoffer Q, and Haigis are commonly used, each with varying accuracy depending on ocular parameters. The SRK formula was among the first empirical methods for IOL power estimation, but it demonstrated significant inaccuracies in eyes with atypical anterior segment anatomy.^[64]^ In the IATS cohort, modern formulas, including Hoffer Q, Holladay 1, Holladay 2, SRK II, and SRK-T, were evaluated, with the median absolute prediction error ranging from 1.3 D (SRK-T) to 3.5 D (Haigis).^[65]^ The SRK-T and Holladay 1 formulas produced comparable results, while the logarithmic model proposed by Dupessey et al attempted to predict axial growth. However, even with personalized a-constant adjustments for sulcus placement, errors were significantly higher in secondary IOL implantation (1.6 D in primary IOLs vs 2.64 D in secondary IOLs, *P* = .035).^[65]^

Given these limitations, different formulas are preferred depending on the child’s age and AL. Irfani et al recommend SRK-T due to its overall accuracy across different pediatric age groups,^[66]^ while Joshit suggests that SRK II provides the most predictable outcomes in eyes with an AL of less than 20 mm.^[67]^ Vasavada challenges these results, claiming that the lowest prediction error was obtained with the SRK-T and Holladay 2 formulas.^[68]^ With the emergence of more advanced formulas, many surgeons are transitioning from third-generation methods to the Barrett Universal II (BU II) formula. A meta-analysis by Hong, including 1781 pediatric cataract eyes, identified Barrett UII, SRK-T, and Holladay 1 as the most accurate for IOL power calculations, with Barrett UII being particularly effective in older children where axial growth has slowed.^[69–71]^ Badakere reports that SRK-T and BU II had the most predictable outcomes in younger children.^[72]^

Beyond these traditional methods, newer-generation formulas continue to improve accuracy. The Intraocular Lens Power Calculator of the European Society of Cataract and Refractive Surgeons integrates 6 calculation models, including BU II, EVO 2.0, Hill-RBF 3.0, Hoffer QST, Kane, and PEARL-DGS.^[73]^ These modern algorithms offer superior predictability in postoperative refractive outcomes, with results comparable to SRK-T, particularly after posterior capsulectomy.^[74,75]^ However, due to continuous axial growth in pediatric eyes and variations in refractive development, no single formula provides a universally accurate solution. Therefore, a combination of predictive algorithms, individualized considerations, and long-term refractive planning remains essential for optimizing pediatric IOL outcomes.

## 5. Age at surgery

Early surgical intervention followed by immediate optical correction and penalization of the dominant eye, achieved through positive defocus (overplus glasses) or replacing the optical correction of the non-amblyopic eye with a plano lens, can be combined with atropine penalization to enhance functional visual acuity.^[76,77]^ The specific duration and frequency should be tailored to the patient’s condition and monitored by an eye care professional. Regular follow-up appointments are essential to assess progress and make necessary adjustments to the treatment plan. Implantation of an artificial lens in infants younger than 6 months carries a high risk of complications, such as higher incidence of visual axis opacities with this treatment compared with aphakia, often requiring reoperation, and is therefore not currently recommended.^[56,78,79]^ Primary intraocular lens implantation in children over the age of 12 months is broadly accepted.^[79–81]^ In most aphakic children, secondary implantation of an IOL is carried out at 1-2 years of age.^[2]^ Kim et al concluded that the optimal age limit is 2 years.^[82]^ Kaplan argued that binocular vision was significantly better in patients undergoing cataract surgery with primary IOL implantation after the age of 2 years.^[83]^ To prevent posterior capsular opacification, a primary posterior capsulectomy (with or without anterior vitrectomy) should be performed at the time of surgery. This procedure is a standard in young children, especially < 3 years of age, as it provides clarity of the visual axis.^[56]^

## 6. Biometry

Cataract surgery in children is more challenging than in adults, as it requires consideration of eye development. Emmetropization involves changes in the refractive structures of the anterior segment and elongation of the eyeball, resulting in emmetropia and full size at 15 years of age.^[84]^ Axial length increases rapidly in the first 2 years of life, after which the growth rate slows, stabilizing at 13 years. Newborns have steep corneas that flatten and thin during infancy, with keratometric power decreasing from approximately 52.0 D at birth to 42.0 to 43.0 D by the end of the first year. Over time, the lens power changes from about 35 D at birth to approximately 19 D. A significant reduction in power occurs by the end of the second year, with a 2-4 D aphakic refractive drop in subsequent years. The anatomical axis length of the pediatric eyeball and keratometry readings are essential for calculating the power of intraocular implants.^[24]^ Given the continuous ocular growth in children, selecting the appropriate IOL requires precise biometry and a tailored approach. Implant selection is made using an accepted formula after determining AL based on corneal refractive power and optical or ultrasound biometry. IOLs implanted in children are usually selected to produce undercorrection; the child is postoperatively hyperopic to counter the expected myopic shift.^[2,24]^

Recommendations regarding target postoperative refraction have evolved over time. Initially, the goal was to achieve emmetropia at the time of surgery. However, due to the high myopic shift, children required IOL exchange. To address this, various predictive models have been developed. The first recommendation for calculating intraocular lens power came from Dahan, who suggested using IOLs with 80% of the power of the emmetropic IOL for children under 2 years and 90% for those aged 2 to 8 years. Lens power reduction should average between 3.00 and 6.00 D. A reasonable approach is to choose lens power based on AL: +28 D at 17 mm, +27 D at 18 mm, +26 D at 19 mm, +24 D at 20 mm, and + 22 D at 21 mm.^[85]^ Enyedi et al provided detailed guidelines with target refractions based on age at surgery: +6 D for a 1-year-old, +5 D for a 2-year-old, +4 D for a 3-year-old, +3 D for a 4-year-old, +2 D for a 5-year-old, +1 D for a 6-year-old, and −1 to −2 D for those older than 8 years.^[86]^ Leksula et al suggested the following IOL corrections: 30% undercorrection in children aged 6 to 12 months, 25% in those aged 1 to 2 years, 20% in 2 to 3-year-olds, 15% in 3 to 4-year-olds, 10% in 4 to 5-year-olds, 2 D undercorrection in children aged 5 to 8 years, and 1 D undercorrection in those aged 8 to 10 years.^[87]^ Vasavada and Chauhan concluded that with an eyeball length of 22.0 mm, the optimal implant power is 22.0 D. With shorter ALs, 2.5 D should be added for every 1 mm decrease.^[88]^ Gordon et al recommend implanting IOLs of 23 to 24.0 D in young patients.^[64]^ Spierer et al suggest a lens power of + 23.2 D to achieve emmetropia at 10 years.^[47]^ The first model for predicting postoperative AL in children after bilateral cataract surgery was developed by Trivedi, marking a significant advancement.^[89]^ In addition to these classical models, recent studies have provided further insights into eye growth in pseudophakic children. Studies based on the IATS cohort have shown that eyeball growth in pseudophakic children is not dependent on age, AL at surgery, the power of the implanted IOL, visual acuity, or the degree of visual impairment.^[90]^ In 2023, Dupessey proposed a new method for calculating IOL power based on age and AL at surgery, using a Napierian logarithm to predict AL growth and target emmetropia at 15 years (previous methods assumed emmetropia at 8 years). Dupessey logarithmic formula estimates refractive error closest to emmetropia. Although further research is needed, this approach shows promise in reducing myopia after pediatric cataract surgery.^[65]^

## 7. Myopic shift

The need for precise biometry and careful IOL selection is directly linked to the phenomenon of myopic shift, which occurs due to axial elongation after infantile cataract surgery. Axial elongation following infantile cataract surgery cannot be fully counterbalanced by corneal flattening, resulting in a myopic shift.^[91]^ In children, primary postoperative hyperopia is intentional, gradually progressing to emmetropia or moderate myopia in adulthood. The younger the patient at the time of surgery, the greater the shift, which persists until at least 8 years of age. There is significant fluctuation in postoperative refractive changes, making it difficult to predict when refraction will stabilize.^[56,92]^ To minimize the need for IOL exchange, the eye should be left undercorrected, with the remaining visual defect corrected using spectacles or contact lenses. In school and preschool-aged children, bifocal spectacles with full correction are recommended due to the loss of accommodation.^[85]^ Weakley et al concluded that to reach emmetropia at age 5, postoperative hypermetropic targets should be + 10.5 D at 4 to 6 weeks and + 8.50 D from 7 weeks to 6 months.^[91]^ Medsinge and Nischal proposed a guideline where children under 1 year receive + 6.00 D, those aged 1 to 2 years receive + 4.00 D, 2 to 3 years + 3.00 D, 3 to 5 years + 2.00 D, 5 to 7 years + 1.00 D, and from 7 years onward + 0.5 D until the age of 11 years.^[3]^ The IATS cohort protocol targeted postoperative hyperopia at + 6.0 or + 8.0 D in infants with an average age of 2.5 months at IOL implantation.^[93]^

## 8. Conclusions

Paediatric cataracts interfere with the development of normal binocular vision, which occurs during the first years of life. Early diagnosis, surgical treatment, correction of refractive defects, and vision rehabilitation are essential. Advances in modern ophthalmic microsurgery and intraocular implant technology have enabled artificial lens implantation in the pediatric population. However, lens selection remains challenging due to unexpected vision defects arising from inaccuracies in biometric techniques, the unpredictability of eyeball growth, and calculation formulas based on normative adult values. Based on current literature, no single biometric formula provides universally accurate results in pediatric IOL power calculations due to the dynamic nature of ocular growth. However, studies indicate that the SRK-T formula remains a widely used and reliable choice, particularly for younger children. The BU II formula has demonstrated superior accuracy in older children due to its advanced vergence-based algorithm and improved AL prediction. Additionally, newer-generation formulas such as Hill-RBF 3.0 and Kane are emerging as promising alternatives, but further validation in pediatric cohorts is needed. Surgical outcomes confirm the effectiveness and safety of implant solutions, enabling vision improvement and facilitating education and daily life participation. Future research should focus on refining pediatric-specific formulas that integrate advanced machine learning algorithms and longitudinal axial growth prediction models to enhance refractive accuracy and long-term visual outcomes.

## Author contributions

**Conceptualization:** Bogumiła Wójcik-Niklewska, Martyna Nocoń-Bratek, Klaudia Szala.

**Data curation:** Martyna Nocoń-Bratek.

**Formal analysis:** Martyna Nocoń-Bratek.

**Investigation:** Martyna Nocoń-Bratek.

**Methodology:** Martyna Nocoń-Bratek.

**Project administration:** Klaudia Szala.

**Supervision:** Bogumiła Wójcik-Niklewska.

**Writing – original draft:** Martyna Nocoń-Bratek.

**Writing – review & editing:** Bogumiła Wójcik-Niklewska, Klaudia Szala.
